# A Curious Case of Recurrent Abdominal Wall Infections

**DOI:** 10.5334/jbsr.2387

**Published:** 2021-02-24

**Authors:** Sara Djelassi, Frederik Vandenbroucke, Martijn Schoneveld

**Affiliations:** 1 UZ Brussel, BE

**Keywords:** laparoscopic cholecystectomy, intra-operative/post-operative complications, (acute) calculous cholecystitis, computerized axial tomography (CT) scan

## Abstract

An 82-year-old male was transferred for an abdominal CT scan for chronic cutaneous fistulation at the level of the right abdominal wall. Previous CT and ultrasound imaging described recurrent collections in the right abdominal wall, requiring CT guided abscess drainage. The abdominal CT scan revealed an abscess in between the internal oblique and transversus abdominis muscle layers of the right flank, with significant fat stranding and loss of the intermuscular fat planes (***Figure 1***). Inside this abscess, we notice a spontaneous hyperdense nodular lesion (Hounsfield Units 130), which doesn’t enhance after contrast injection (***Figure 1*** arrow). Looking back at the previous CT scans we discern the presence of this hyperdense lesion, which tends to migrate over time over a small distance along the abdominal wall (***Figure 2 A–D*** arrow). We can trace this back on the numerous previous scans, with different local tissue reactions over time. The first performed CT 8 years prior reveals a perforated calculous cholecystitis, containing multiple cholecystolithiases with the same density as our previously mentioned hyperdense lesion (***Figure 3*** arrow). Thus, raising the suspicion of a biliary origin of this corpus alienum.

**Teaching point:**

Spilled gallstones during laparoscopy may lead to late abscess.

## Case report

An 82-year-old male was transferred for right abdominal wall chronic cutaneous fistulation.

**Figure 1:**
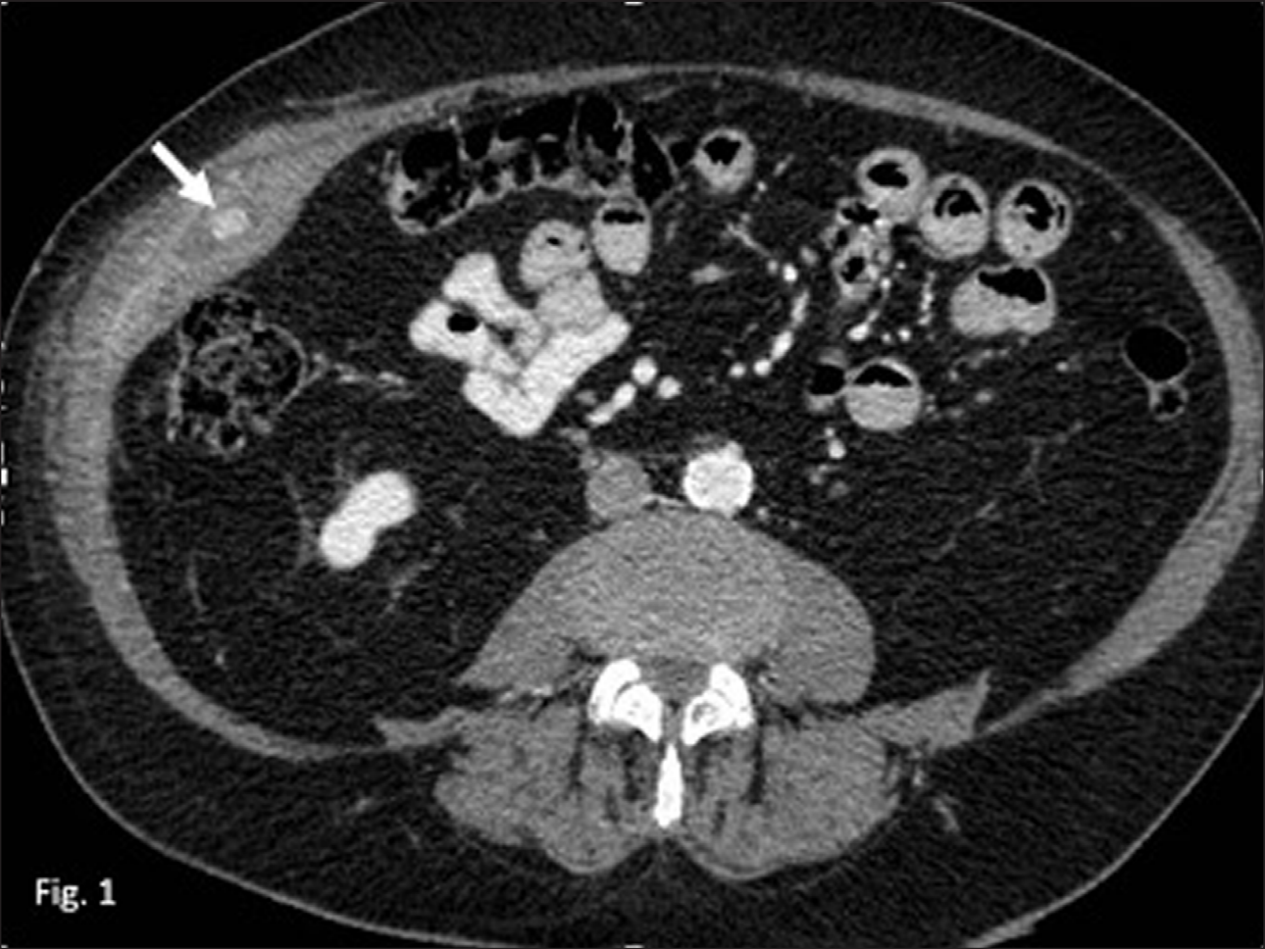


Clinical and imaging records were positive for recurrent abscess in the right abdominal wall, requiring drainages. Abdominal computed tomography (CT) evidenced a new abscess in between the right internal oblique and transversus abdominis muscle layers, with fat stranding and loss of the intermuscular fat planes. Within this collection, there was a hyperdense (130 Hounsfield Units) centimetric nodular structure (***Figure 1***, arrow).

**Figure 2:**
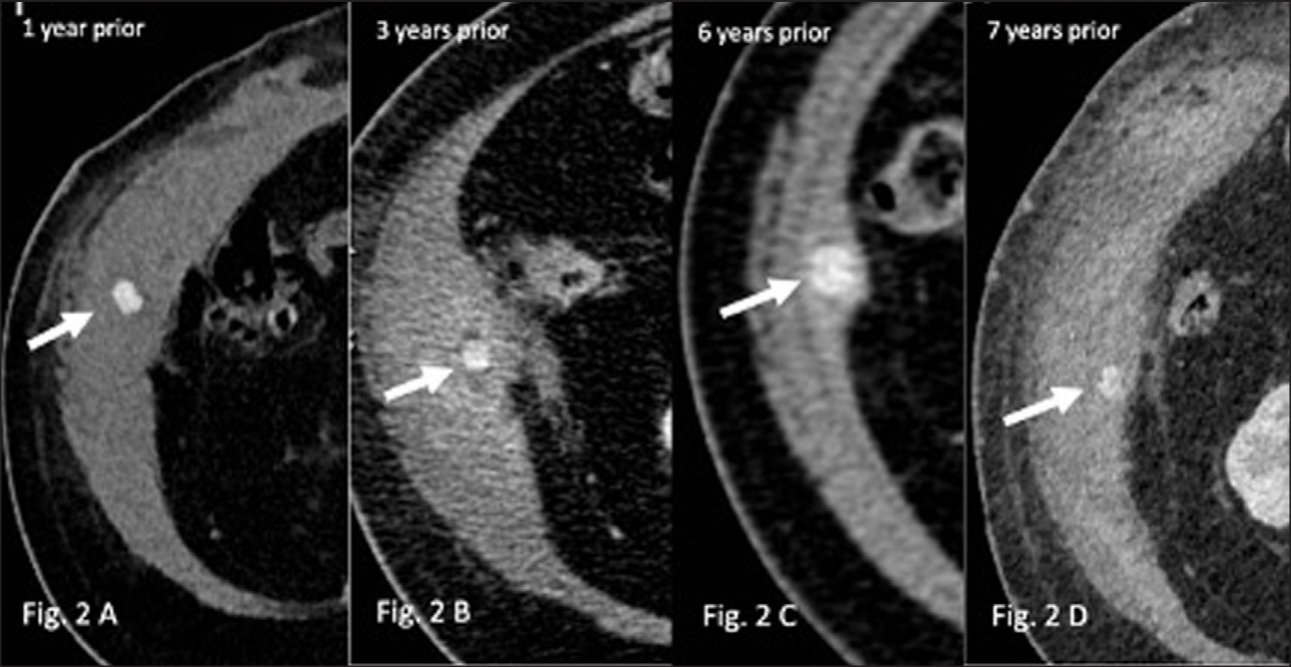


**Figure 3:**
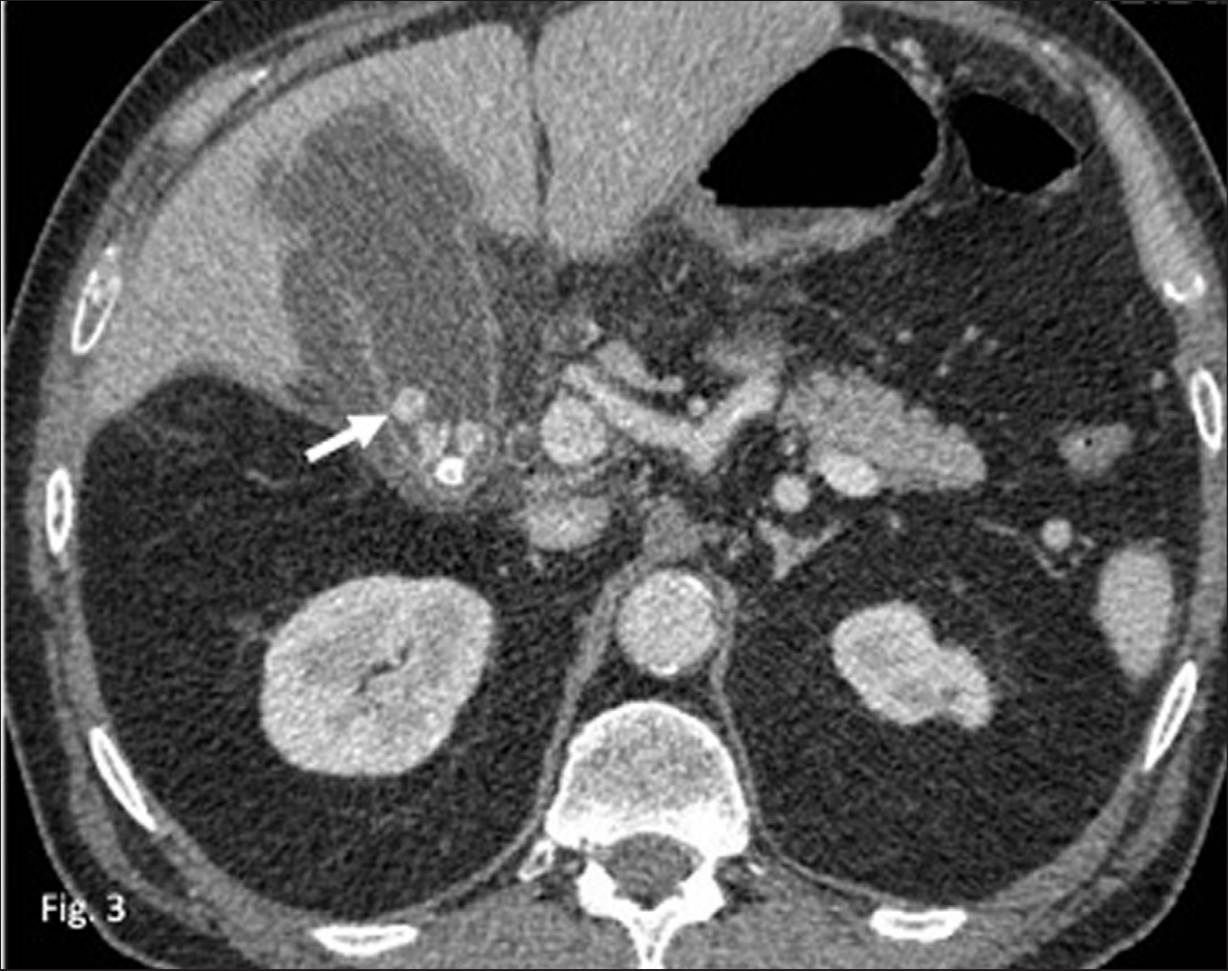


A first CT, performed eight years earlier, at the time of a perforated cholecystitis, evidenced multiple gallstones with similar density and size as the actual abdominal wall structure (***Figure 3***, arrow), thus raising the suspicion of a migrated gallstone. Looking back at the previous CTs we were able to identify the same structure, though it tended to slightly migrate with time over a small distance along the abdominal wall (***Figure 2 A–D***, arrows). A revision fistulectomy revealed the presence of a corpus alienum in the right abdominal wall, and histological examination confirmed its biliary origin. The patient recovered well and didn’t present any surgical site or abdominal complications since.

## Comment

Laparoscopic cholecystectomy is accepted as the gold standard for acute gallbladder diseases. The most common cholecystectomy-related complication is an iatrogenic perforation of the gallbladder. Spillage of stones can occur during dissection of the gallbladder or tearing with grasping forceps, responsible for intraperitoneal dislodgement.

Less frequently, spillage may also arise during the extraction of the gallbladder through one of the trocar sites, where the retrieval bag can rupture and lead to contamination of the port site. An acutely inflamed or necrotic gallbladder is a predisposing factor for iatrogenic perforation and spillage.

Radiologists should consider spilled stones as a potential source of recurrent abscesses in any patient presenting months or even years after a laparoscopic cholecystectomy [1].

While uncommon, these stones may lead to late complications, which can be a diagnostic challenge and cause significant morbidity. An hyperdense structure on CT within an abdominal or parietal collection should raise the suspicion of spilled gallstone or even appendicolith [2], depending on the patient’s medical history. Of note, non-opaque stones missed on CT can be diagnosed by ultrasound.

## References

[1] Zehetner J, Shamiyeh A, Wayand W. Lost gallstones in laparoscopic cholecystectomy: All possible complications. Am J Surg. 2007; 193(1): 73–78. DOI: 10.1016/j.amjsurg.2006.05.015 17188092

[2] Lambo A, Nchimi A, Khamis J, Khuc T. Retroperitoneal abscess from dropped appendicolith complicating laparoscopic appendectomy. Eur J Pediatr Surg. 2007; 17(2): 139–41. DOI: 10.1055/s-2007-965006 17503311

